# A Query Result Merging Scheme for Providing Energy Efficiency in Underwater Sensor Networks

**DOI:** 10.3390/s111211833

**Published:** 2011-12-20

**Authors:** Yunsung Kim, Soo-Hyun Park

**Affiliations:** 1 Korean Minjok Leadership Academy, 1300, Sosa-ri, Anheung-myeon, Hoengseong-gun, Gangwon-do, 225-823, Korea; E-Mail: jkkim008@hanmail.net; 2 The Graduate School of Business IT, Kookmin University, 861-1, Jeongneung-dong Sungbuk-gu, Seoul, 136-702, Korea

**Keywords:** underwater sensor network, query management, query result merging, relational sensor network database, conditional query, periodic query

## Abstract

Underwater sensor networks are emerging as a promising distributed data management system for various applications in underwater environments, despite their limited accessibility and restricted energy capacity. With the aid of recent developments in ubiquitous data computing, an increasing number of users are expected to overcome low accessibility by applying queries to underwater sensor networks. However, when multiple users send queries to an underwater sensor network in a disorganized manner, it may incur lethal energy waste and problematic network traffic. The current query management mechanisms cannot effectively deal with this matter due to their limited applicability and unrealistic assumptions. In this paper, a novel query management scheme involving query result merging is proposed for underwater sensor networks. The mechanism is based on a relational database model and is adjusted to the practical restrictions affecting underwater communication environments. Network simulations will prove that the scheme becomes more efficient with a greater number of queries and a smaller period range.

## 1. Introduction

A wireless sensor network (WSN) is a wireless network of spatially distributed autonomous devices (sensor nodes) using sensors to cooperatively monitor physical or environmental conditions, such as temperature, sound, vibration, pressure, motion or pollutants, at different locations. [1–3] Each sensor node in a sensor network has functions to sense physical or environmental conditions, to process sensed data, and to send or receive the sampled data or processed results. Sometimes, a sensor node might be equipped with actuators to control itself or the environment.

Recently, applications of wireless sensor networks to underwater environments arose as a promising division of research among scientists due to the systems’ various application possibilities [4–6]. Underwater sensor networks are designed for applications like pollution monitoring, disaster prevention, strategic surveillance, oceanographic data collection and offshore exploration. Unmanned vehicles with several underwater sensors can also be utilized for underwater resource investigation and collaborative underwater data gathering monitoring tasks [4]. Acoustic arrays and sensors can also be deployed for underwater ecosystem monitoring, in which transceivers can detect and track the number, health and location of aquatic creatures [7,8].

One important challenge with underwater sensor networks is enabling underwater communications. Wireless sensor network communication on land is conducted mostly through radio frequency (RF) wave propagation, taking advantage of its speed, long range and reasonable energy expenditure [9]. For underwater communications, however, RF waves are inappropriate due to several reasons such as high dissemination rate, immense propagation power [10,11], and high exposure to communication interference due to low and restricted transmission bandwidth [12,13]. Therefore, efforts have been undertaken to apply acoustic waves as a means for underwater sensor network communication [12,14].

Underwater acoustic wireless sensor networks, however, still display severe flaws that hinder their utilization for comprehensive and exhaustive communication tasks. First, acoustic waves are much slower than RF waves and can travel at only 1.5 km/s, so real-time data transmission and time synchronization are very difficult [4]. Also, pulsing an acoustic signal underwater requires more transmission power than for terrestrial RF signals [4]. Lastly, due to the high bit error rate, a greater amount of data transmission incurs a proportionally large amount of data failures [14].

Therefore, we face an urgent need to reduce the amount and frequency of data transmissions, especially in underwater circumstances. Some studies on data storage sought to reduce redundant data conveyance by allowing users to selectively acquire data by querying the network [15]. One of the two major approaches to data storage in sensor networks is the “warehouse” approach, in which all sensor data is collected into a large, central database from which users can acquire their data of interest [15]. Another approach is the distributed approach, in which each distributed devices such as sensor nodes can act like a database and users query each node for data [16]. The distributed approach effectively reduced the redundant network traffic caused by the warehousing system [17]. With recent enhancements in ubiquitous technologies, more smart devices (smart phones, smart dust, smart tabs *etc.*) are expected to be utilized for real-time and user-friendly seismic monitoring, equipment monitoring and remote controlling, leak detection, and support for robot swarms through this approach [18].

However, such a query-based sensor network management strategy can only be effective when a few users are online. When many users query the whole network through ubiquitous data management systems, responses to some queries may contain the responses for other queries, in which case redundant query responses may engender problematic waste of energy and traffic and cause high possibility of bit errors [19]. Energy is extremely wasted, especially when the node has to transmit the headers attached to every additional message it sends. To reduce excessive data transmissions, query aggregation and other researches on enhancing query processing were conducted [17,19], but there still is a great lack of studies on managing condition-based queries (request for data that satisfies query conditions).

In this paper, we suggest a multiple query result merging scheme as a practical solution for the above problems in underwater sensor network data transmissions. The proposed framework is a technique for providing data on-demand from an underwater sensor network to multiple-users simultaneously in an energy-efficient manner. Our paper’s main focuses are:

1. A relational database model view suitable for underwater sensor network applications
  A relational underwater sensor network database model view is proposed. In conventional sensor network database models, time synchronization is guaranteed. Because time synchronization is very difficult in underwater sensor networks, however, we employ the “current relation”, a single tuple relation of current sensor data, in our model. The queries request periodic data transmissions from these current relations. This model view allows us to access the sensor data in a sensor network using relational database concepts.
2. The Query Managing Theorem and Period & Duration Management schemes
  We give a theoretical account of queries and the basic principles of our query result merging mechanism. On these grounds, we provide an efficient query result merging mechanism. We also provide a new methodology for managing a number of continuous queries with different periods and durations.
3. The comprehensive query management framework and a new payload format
  A complete query management framework is thoroughly proposed. The management framework is categorized into four phases: Query Transmission Phase (QTP), Query Processing Phase (QPP), Query Response Phase (QRP), and Response Distribution Phase (RDP). A new payload format suitable for our proposed query management framework is devised and introduced.

The rest of this paper is organized as follows: Section 2 reviews the current research *status quo*, Section 3 explains the basic network and query models in discussion, Section 4 provides a mathematical approach to query result merging and its theorems, Section 5 comprehensively explains the four steps of query management framework, and Section 6 sets up a simulation model for performance evaluation, simulates the proposed framework and discusses the results. Section 7 reorganizes our contributions, suggests future work and concludes the whole paper.

## 2. Related Work

Several projects have introduced diverse mechanisms for distributed data querying, some of which include the Cougar system and the TinyDB. The Cougar system is a query processing mechanism that consists of three layers: front end, cluster leader, and the cluster node [16]. Each cluster node keeps its sensor data in the form of a time-series [20]. When the user sends a query to the network, the queries are transmitted to the front end, a cluster leader, and eventually delivered to the cluster node, which transmits the queried data in the reverse manner [16]. The TinyDB is a query processing system for extracting information from a network of TinyOS sensor nodes. When the user requests certain data with an SQL-like query language, the nodes take readings from the current node and merge it with sub-aggregates from the subtree, returning the node’s aggregate data up the tree [21].

Both of these systems, however, lack query optimization schemes, and thus, are very vulnerable to heavy network traffic caused when many users submit multiple queries simultaneously. To alleviate network traffic and reduce energy waste, many researchers have studied data aggregation schemes as in the directed diffusion methods studied by [22] or the data centric routing schemes proposed by [23]. Several studies on effective data routing protocols and localization architectures were conducted by [19,22,24–27] as well. However, these researches were focused on efficient data handling without much focus on query or query result managements.

Some of the most closely related works on query management are [19,25]. Document [19] suggests a query aggregation mechanism for location-based queries. In [19], a multi-layer query aggregation method was introduced, in which the query manager aggregates queries and sends the merged query to each of the access points, where the queries are distributed among the target nodes. This project, however, only takes snap shot queries into account and disregards continuous queries (requests for data transmissions over a certain period of time).

In [28], a concept of condition-based query merging similar to our approach is introduced. The project, however, is immature and lacks theoretical grounds. Furthermore, in [19,28] no management methodologies for continuous queries are provided and their naïve approach to merging queries bears significant flaws and inefficiencies that are addressed and resolved in our paper. The project also lacks a generalized query processing scheme.

## 3. Underwater Acoustic Sensor Network Models and Query Models

In this section, we explain the informational background of underwater sensor networks. Then we explain our network database view and query model.

### 3.1. Underwater Acoustic Sensor Network Backgrounds

**Figure 1.**
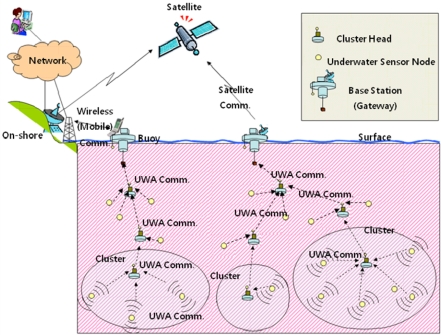
Cluster-based underwater acoustic sensor network model.

As explained in the introduction, our main focus is to provide practical and efficient data management framework for underwater sensor networks. An underwater sensor network usually consists of sensor nodes, clusters and base stations. Each terminal node transmits the sensor data toward the cluster header. The cluster header collects sensor data from a number of terminal nodes and tosses the packet up the tree. Using certain routing mechanisms, these intermediate nodes transfer the packet to each other and eventually deliver the packet to a base station placed at the surface, where the acoustic data signals are transformed into corresponding RF signals and transmitted to the user applications [23,24]. Figure 1 shows the underwater acoustic sensor network model.

One major disparity between the common RF based sensor networks and underwater acoustic sensor networks is time synchronization. As explained in the introduction, low signal propagation speed and limited human accessibility to underwater sensor nodes makes time synchronization very difficult. In our network model, therefore, sensor nodes are assumed to execute queries individually without any agreement on time, and the following network database and query models are modified on such basis.

### 3.2. Relational Underwater Sensor Network Database Model

For better sensor network data management, many attempts have sought to apply database models to sensor networks. Relational databases serve as useful tools for systematized sensor network data management because of their simple data structure, suitability for queries, and simple manipulation languages [20]. In our approach we follow a similar concept.

Any sensor network can be mapped to a relational database model. In our data model, each sensor node represents a relation, where each sensor and node ID is the relational attribute and the sensor value range is the attribute domain. Each node memory contains a number of ordered lists of sensor data at a certain instant. Each list is mapped to a tuple in a sensor node relation, and the collection of these relations is the sensor network database.

**Figure 2.**
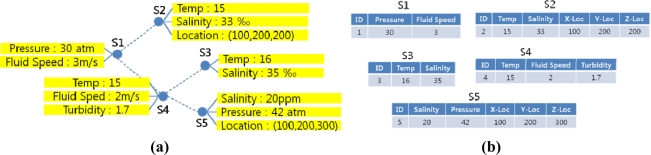
Example of a relational underwater sensor network database model. The sensor network in **(a)** can be mapped to a relational database model in **(b)** where each of the sensors is mapped to one of the current relations.

In some of the relational sensor network database models, a sensor node keeps a partial history of recent sensor data. In these models old tuples are constantly updated and replaced by new ones, and queries extract data only from the remaining tuples. However, because underwater signal propagation is significantly vulnerable to water environments, there is an ample possibility that the requested data has already been deleted from the node relation before the query reaches the node. Also, difficulties in time synchronization make sensor node data timeline ineffective for underwater acoustic sensor network applications. Therefore, our network database model employs a single-tuple relation of current sensor data called “current relation”. The current relation is updated whenever queries are assigned. For example, the sensor network in Figure 2(a) can be mapped to a corresponding relational database in Figure 2(b).

### 3.3. Query Models

Users frequently request on-demand data transmissions using queries. Most of the queries are condition-based selection queries (requests for data transmissions under certain conditions), and projection queries (requests for transmissions of certain attribute values of sensor data). Users can also demand periodic data transmissions and stop the responses after a certain period. Based on these observations, all queries can be represented by the following model:

$$Q=o{|}_{\left(S,\;D\right)}^{T}$$

where:

- O = query operation, a one-time data request (a finite combination of selection and projection queries: *o* ∈ *Span* <*σ*,Π>)
- T = data reply period in terms of unit time
- S = query reply start time represented by the number of time units after the nodes query set start time (for a detailed explanation of query set start time, refer to Section 5.3.1.)
- D = query deadline also represented by the number of time units after the query set start time

The queries’ temporal information (period, start time and deadline) is arranged in terms of a unified atomic time unit. The specific length of the time unit is determined prior to all query operations. The following are two examples of our query model.

Example (1)

*Q*_1_: Transmit the pressure value for sensor S5 every 2 s for 30 s (unit time: 1 s).

$$Q_{1}={\Pi_{\mathrm{pressure}}\left(\sigma_{\mathrm{ID}=S_{5}}\left(S\right)\right)\;|}_{\left(0,\;30\right)}^{2}$$

Variable S refers to a dummy relation variable for a sensor node relation.

Example (2)

*Q*_2_: Transmit the pressure and temperature values every 3 s for 15 s if the temperature is above 20 degrees and the depth is greater than 50 meters (unit time: 1 s).

$$Q_{2}={\Pi_{\mathrm{pressure},\mathrm{temp}}\left(\sigma_{\mathrm{temp}>20\;\wedge \;z-\mathrm{loc}>50}\left(S\right)\right)\;|}_{\left(0,\;15\right)}^{3}$$

When we refer to a “query”, by definition it refers to the periodic data transmission request over a certain period of time. We hereby note, however, that in the following sections, the notation “query” may also frequently refer to a one-time data request in the continuous transmission task at some moment (or simply, a query operation), as long as no specific temporal information is mentioned and there is no ground for confusion. For example, “responses to queries *q*_1_ and *q*_4_” would refer to the relations resulting after the two query operations are executed at a given time frame.

## 4. Theoretical Approach to Query Result Merging

In this section, we explain and prove two major theorems useful for query result merging along with important definitions.

### 4.1. The Query Reduction Principle

In this section, we explore the properties of queries and define query result merging. Following is a definition of a well-defined query.

**Definition 1.** Well-Defined Query

Let us denote that condition *C* “refers” to a set of attributes *A* when all the data fields involved in condition *C* is contained in set *A*.

Query:

$$q=\Pi_{A_{n}}\left(\sigma_{C_{n}}(\cdots \left(\Pi_{A_{1}}\left(\sigma_{C_{1}}(S)\right)\right)\right)$$

is well-defined if condition *C_i_* refers to a subset of attribute set *A_i−1_* for every positive integer *1 < i* ≤ *n*.

This definition is reasonable since selection *σ*_*C*_*j*__(…) would be examining non-existent attributes if *C_i_* does not refer to the attributes in *A_i−1_* because projection Π_*A*_*i*−1__(…) would leave out the attributes referred in *C_i_*. Otherwise, all queries can be answered. The following theorem shows how all queries can be reduced to a simple form. Without loss of precision, we ignore the query temporal information for now.

**Theorem 1.** Query Reduction Principle

Every well-defined query:

$$q=\Pi_{A_{n}}\left(\sigma_{C_{n}}\left(\cdots \left(\Pi_{A_{1}}\left(\sigma_{C_{1}}(S)\right)\right)\right)\right)$$

can be reduced to the form:

$$q=\Pi_{A}(\sigma_{C}(S))$$

where *A = A_n_* and $C=\overset{n}{\underset{i=1}{\wedge }}C_{i}$

**Proof**

We prove this theorem through mathematical induction.

We first examine when *n = 2*. If *C* refers to *A*:

$$\sigma_{C}\left(\Pi_{A}\left(S\right)\right)=\Pi_{A}\left(\sigma_{C}\left(S\right)\right)$$

since selecting pairs and restricting them to set *A* is equivalent to doing the reverse as long as *C* refers to *A*. Therefore:

$$q=\Pi_{A_{2}}\left(\sigma_{C_{2}}\left(\Pi_{A_{1}}\left(\sigma_{C_{1}}\left(S\right)\right)\right)\right)=\Pi_{A_{2}}\left(\Pi_{A_{1}}\left(\sigma_{C_{2}}\left(\sigma_{C_{1}}\left(S\right)\right)\right)\right)$$

Note that:

$$\Pi_{A_{2}}\left(\Pi_{A_{1}}\left(X\right)\right)=\Pi_{A_{2}}\left(X\right)$$

because *A*_2_ ⊂ *A*_1_ and that:

$$\sigma_{C_{1}}\left(\sigma_{C_{2}}\left(X\right)\right)=\sigma_{C_{1}\wedge \;C_{2}}\left(X\right)$$

because the resulting tuples satisfy both C_1_ and C_2_. Therefore, *q* = Π*_A_* (σ_C_(*S*)) for *n = 2*, where *A = A_2_* and *C* = *C*_1_ ∧ *C*_2_.

Assume that the principle is true for *n = k*. Then for *n = k + 1*:

$$q=\Pi_{A_{k+1}}\left(\sigma_{C_{k+1}}\left(\cdots \left(\Pi_{A_{1}}\left(\sigma_{C_{1}}(S)\right)\right)\right)\right)$$

Since the nested operation: $\Pi_{A_{k}}\left(\sigma_{C_{k}}\left(\cdots \left(\Pi_{A_{1}}\left(\sigma_{C_{1}}(S)\right)\right)\right)\right)$ equals *q* = Π*_A_*_′_ (σ*_C_*_′_(*S*)), where *A’= A_k_* and $C\prime =\overset{k}{\underset{i=1}{\wedge }}C_{i}$, *q* can also be reduced to the form:

$$q=\Pi_{A_{k+1}}\left(\sigma_{C_{k+1}}\left(\Pi_{A^{\prime }}\left(\sigma_{C^{\prime }}(S)\right)\right)\right)$$

as examined in the case when n = 2. Hence:

$$q=\Pi_{A_{k+1}}\left(\Pi_{A^{\prime }}\left(\sigma_{C_{k+1}}\left(\sigma_{C^{\prime }}(S)\right)\right)\right)=\Pi_{A}(\sigma_{C}(S))$$

where A = A_k+1_ and $C=\overset{k+1}{\underset{i=1}{\wedge }}C_{i}$.

Query Reduction Principle is meaningful since it implies that all queries can eventually be reduced to its simplest forms that are much easier to deal with. On this foundation, we introduce our new query result merging methodology.

### 4.2. Query Result Merging Methodology

Here, we define query result merging and discuss its significance.

**Definition 2.** Query Result Merging

Let Σ = {*q*_i_} be a set of queries, where:

$$q_{i}=\Pi_{A_{i}}(\sigma_{C_{i}}(S))$$

Also, given a current relation *S*, let Σ*_S_* be a set of queries in Σ whose conditions are satisfied by the tuple in *S*. Then the relation defined by:

$$M_{\Sigma }(S)=\Pi_{\cup_{q_{i}\in \Sigma_{S}}A_{i}}(S)\;\;(A_{i}:\;\text{the set of projection attributes of query}\;q_{i}).$$

is called the merged query result for the query set Σ and the process of producing the merged query result relation is called “query result merging”.

Logically, it is reasonable to call this “query result merging” because the process extracts only the data fields requested by the queries whose conditions are satisfied by the current relation *S*. Therefore, the resulting relation fully contains the requested data in the most compact manner.

Unlike in [28], where query conditions and requested data attribute sets were merged, we do not attempt to merge queries. Merging queries into a single query is dangerous because when query conditions and projection attributes are merged, the merged query would indistinctively request all data attributes, even for the queries whose conditions are not satisfied. Therefore, instead of incurring unnecessary data transmissions by query merging, we apply a more robust concept of query result merging. The following section explains the complete query management framework for underwater sensor networks in detail.

## 5. The Query Management Scheme

In this section, we briefly explain our framework settings and illustrate our proposed query management framework in detail.

### 5.1. System Architecture

**Figure 3.**
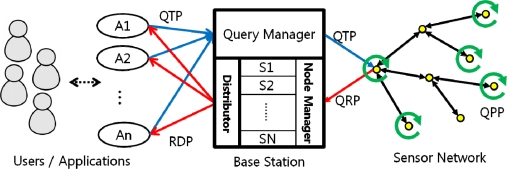
System architecture for the query management scheme.

Figure 3 briefly illustrates our system architecture, which consists of four major components: the users, the user applications, the Base Station and the sensor network. Below is a brief description of each component.

**Figure 4.**
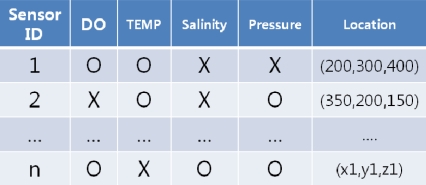
Example of a network dictionary possessed by a BS.

1. The Users/Applications
  Each user uses an application to access underwater sensor networks. The users can send queries and receive query results through the base station with the user applications.
2. The Base Station (BS)
  In our system architecture, the base station plays a crucial role in organizing queries into sets, receiving sensor data from each node, reorganizing the query results, and transmitting the data to each of the applications. Since the BS assumes an extensive role, it is divided into three units, each of which is enumerated below. The BS has a comprehensive network metadata called a “network dictionary” that contains the information of sensors attached to each node and the location of node. With the network dictionary, the Base Station selects the “target nodes” for each query—the nodes that collect the data fields requested by each query. Figure 4 is an example of a network dictionary. The BS is provided with infinite processing/transmitting power.
  - The Query Manager (QM)
    The QM receives queries from the users up to as much as a node can process at a time, arranges them into a query set, and routes the set to targeted sensor nodes. The network dictionary is used to select the target nodes.
  - The Node Manager (NM)
    The NM receives the query result tuples from the sensor nodes. Using the time stamps on the result tuples, the NM generates a timeline of queries to determine which queries are responded at which moment. It then generates the final response tuple that has a number label to clarify its time sequence, and passes the differently labeled tuples to the Distributor.
  - The Distributor
    The distributor receives the final tuples from the NM and sends the arranged query results to the user applications.
3. Sensor Nodes
  Each sensor node receives queries and merges the query results. It sends back result tuple to the BS.

Our query management scheme is divided into four phases: the Query Transmission Phase (QTP), the Query Processing Phase (QPP), the Query Response Phase (QRP), and the Response Distribution Phase (RDP). The following subsections provide detailed explanations for each phase.

### 5.2. The Query Transmission Phase (QTP)

**Figure 5.**
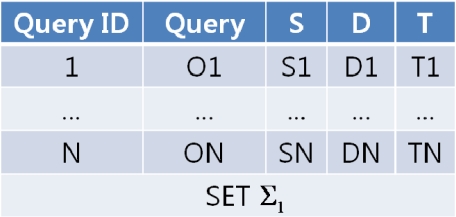
Example of a query set sent from the BS to a node.

When users send queries to the QM, the QM sequentially assigns an ID to each query and goes through the network dictionary to determine which nodes to forward the queries to. The QM generates a collection of queries targeted to each sensor node (called a “query set”) and routes these query sets to each of the target nodes. Queries are collected as much as each node can transmit at a time. If there aren’t as many queries, the QM can periodically formulate a query set and transmit it to the network. In Figure 5, *S_i_*, *D_i_*, *T_i_* (i = 1, 2,…, N) denote the query’s start time, deadline and response period.

### 5.3. The Query Processing Phase (QPP)

The QPP is the core of our management scheme. In this phase, each sensor node receives its query set and merges the query results to generate the response message.

#### 5.3.1. Query Set Start Time

When the node receives a query set Σ*_i_*, it automatically assigns a query set start time *I_i_*, based on the time indicated by each node clock. The query set start time is the zero time point for all the temporal information within the set. The set start time for the first query set received by the node is 0. For example, let’s say the QM sent two query sets, first Σ_1_ then Σ_2_, to a node as the following:

$$\begin{array}{l} \begin{array}{c} \Sigma_{1}=\left\{q_{1},\;q_{2},\;q_{3},\;q_{4}\right\}\;\text{and}\;\Sigma_{2}=\left\{q_{5}\right\}\\ q_{1}=o_{1}{|}_{\left(0,\;10\right)}^{5},\;q_{2}=o_{2}{|}_{\left(0,\;12\right)}^{2},\;q_{3}=o_{3}{|}_{\left(0,\;12\right)}^{3},\;q_{4}=o_{4}{|}_{\left(0,\;12\right)}^{6},\;q_{5}=o_{5}{|}_{\left(0,\;9\right)}^{5} \end{array} \end{array}$$

**Figure 6.**
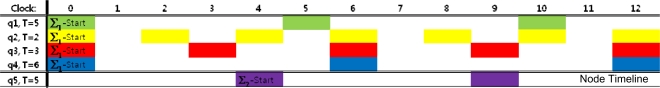
Timeline of queries sent to a node. The numbers indicate the time of the readings from the node clock. Each colored box indicates query execution.

Figure 6 is a timeline of the queries sent to the node. The numbers on the first row indicates node clock and the colored boxes indicate query execution. For instance, when the node clock indicates 6, queries *q*_2_, *q*_3_ and *q*_4_ must be conducted. In this figure, *I_1_* is 0 and *I_2_* is 4. The queries in Σ_2_ are executed every 5 time units after the query set start time.

#### 5.3.2. Period and Duration Management Theorem

Given the list of queries, the node scans through the queries and their temporal information to select which queries to respond to at each moment. The following theorem provides a method of selecting queries for generating their responses at a specific time point.

**Theorem 2.** Period and Duration Management Theorem

Let Σ*_t_* denote the set of all queries arrived at a node up to time *t*. Then, the set of queries waiting to be responded at t is defined by:

$$\Lambda_{t}=\left\{q_{i}\in \Sigma_{t}|t\equiv \left(I_{i}+S_{i}\right)\;\;\text{mod}\;\;T_{i},\;\;t\;\;\leq \;\;\left(I_{i}+D_{i}\right)\right\}$$

where *S_i_*, *D_i_*, *T_i_* and *I_i_* are the start time, deadline, response period, and query set start time for query *q_i_*.

**Proof**

Because the query set start time is the zero point for the queries in the set, the first execution time for query *q_i_* is *t* = *I_i_* + *S_i_*. Query *q_i_* is replied every *T_i_* since the first execution up to the deadline, which means queries are executed at *t* ≡ (*I_i_* + *S_i_*) mod *T_i_* while *t ≤ I_i_* *+D_i_*. Therefore, the merged query result at time *t* is *M*_Λ_*_t_*(S).

#### 5.3.2. The Message Payload

**Figure 7.**
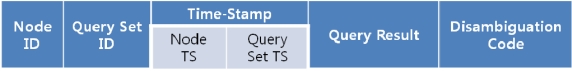
Response message payload format at the application level.

With the query result tuple, the node creates a response message. Figure 7 is the message payload format for the node’s application level. The data fields in the payload are explained below.

1. Node ID: The node ID is the ID of sensor node that sent the response message.
2. Query Set ID: The query set ID is the set ID of the most recently arrived query set. This field is used to identify the query set start time for different query sets.
3. Time-Stamp: This field indicates when the response was made. Using the time-stamp, the Node Manager can generate a query timeline to determine which queries are executed at each moment. The Query Set time-stamp is added to adjust to message transmission failures, as explained in the Section 5.3.1.
  - Node-Time Stamp: The node time-stamp is the time indicated by the node clock. The zero time point for the node clock is the query set start time of the first query set.
  - Query Set Time-Stamp: The query set time-stamp represents the number of unit time after the start time of the most recently arrived query set. For example, if the latest query set arrived at Node TS = 14 and the current time is Node TS = 16, then Query Set TS = 2.
4. Disambiguation Code (DC): This is a binary code included in case there is an ambiguity caused by multiple queries requesting the same data attributes under different query conditions. Each of the ambiguous queries is sequentially represented by each bit of the code. If the query condition is satisfied, the digit is set to 1; else the digit is clear.
  For example, assume that the node has to execute the following three queries:
  $$\begin{array}{c} q_{2}=\Pi_{\mathrm{temp}}(\sigma_{\mathrm{pressure}>20}(S))\\ q_{3}=\Pi_{\mathrm{temp},DO}(\sigma_{\mathrm{pressure}<20}(S))\\ q_{4}=\Pi_{\mathrm{DO}}(\sigma_{\mathrm{temp}<30}(S)) \end{array}$$
  If the sensor data readings are: *pressue* = 30, *temp* = 20, the node sends both the temperature and the DO values in response to *q*_2_ and *q*_4_. However, with only the two field data, the Node Manager cannot recognize whether the pairs are responses to query *q*_3_ (which also demands both the temperature and the DO values) or to queries *q*_2_ and *q*_4_. Therefore, a three-digit disambiguation code “101” is added to indicate which query conditions (*q*_1_ and *q*_3_ in our case) are satisfied.

**Figure 8.**
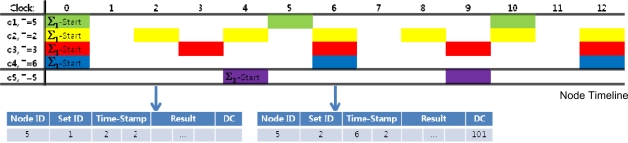
Examples of response messages at two different points in time. The first response message has no disambiguation code because there is no possible ambiguity.

Figure 8 is an example of two response messages at different time frames for a sensor node whose ID is 5. Notice that no disambiguation code is required for the first response message because there is only one query, which indicates no possibility of ambiguity.

#### 5.3.3. Robustness against Transmission Failures

Due to their hazardous communication environment, underwater sensor networks are highly exposed to communication failures. Especially when response messages are missing at the start of a new query set as in the following examples, a single time-stamp may cause critical mishaps to the NM when distinguishing query set start time.

**Figure 9.**
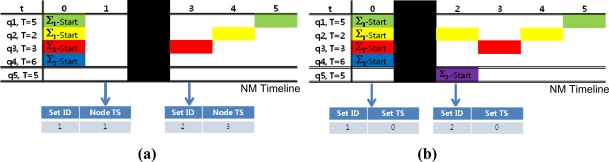
Examples of how single time stamps can be misleading in case of communication failures. **(a)** The response message only contains node time stamp. **(b)** The response message only contains set time stamp.

In Figure 9(a,b), the original query set start time for Σ_2_ is t = 2, but a response message is missing around the query set start time. In Figure 9(a), a response message is missing at t = 2. With only node time stamps as a time guide, the NM will mistake t = 3 as the set start time for Σ_2_ because this is the first time Σ_2_ appears in the NM. Similarly, in Figure 9(b) the message is missing at t = 1. The node time stamp is also be misleading since the NM would identify t = 1 as the start time for Σ_2_ because Σ_2_ first appears after the set time stamp changes from 0. Therefore, single time stamps are significantly vulnerable to communication failures and can influence the whole query management process.

With both the node and query set time stamps as time indicators, the NM can identify the query set start time by tracing the changes in the set ID and the node time stamp. The NM can also overcome intermittent message blackouts and keep track of exact time sequences.

### 5.4. The Query Response Phase (QRP)

In this phase, responses are transmitted from the nodes to the NM, which puts a number label to each of the tuples and passes the pairs to the Distributor. When the NM receives a response message, the NM can generate a query timeline (similar to the timelines in the above examples), using the time information elucidated by the response message, the start time for each query set, and each query’s temporal information. It can also distinguish the sequence of the response tuples. The NM generates the final response tuple by extracting the requested data fields from the response message and putting the query ID, node ID, and a sequence number label to each of the extractions, and passes the final tuple to the Distributor. The final tuple is organized by the Distributor and sent to each user. Result tuples can be extracted by using the Data Extraction Theorem. The proof of the theorem is obvious, so it is not provided.

**Theorem 3.** Data Extraction Theorem

The NM can extract the data requested by query *q_i_* from the result tuple by the following operation:

$$q_{i}(S)=\Pi_{A_{i}}(T)$$

where *A_i_* is the data attribute requested by *q_i_* and *T* is the result pair relation.

**Figure 10.**
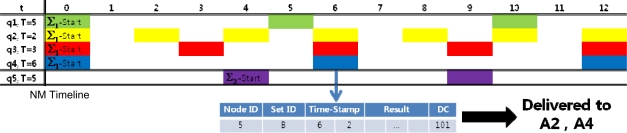
An example of message processing at the QRP. Analyzing the disambiguation code, the NM can determine that the queries should be sent to applications A2 and A4.

**Figure 11.**
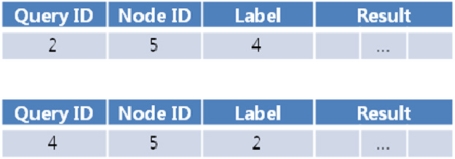
Two examples of response messages delivered to the Distributor. **(a)** is delivered to A2 and **(b)** is delivered to A4.

Figure 10 is an example of message processing at this phase. *A_i_* is the user application that sent the query *q_i_*:

In this example, the NM generated a query timeline. When the NM receives a query response message from a node (sensor node 5 in this case), the node can identify which users to send the queries to. In our case, the disambiguation code is 101, which indicates that there are three ambiguous queries and the query results should be transmitted to the user applications for the first and last of the three (A2 and A4). In the final response messages generated for user application A2, the sequence number label would read “4” since it is the fourth response to query q2. Similarly, the sequence number label for user A4 would read “2”. Final response messages are sent to each distributor would look like Figure 11.

### 5.5. The Query Transmission Phase (QTP)

This is the last phase of the query management scheme. In this phase, the Distributor receives queried data from the NM, and each query response is sent to the appropriate user applications. According to the users’ demands, the Distributor can organize the queried data into time sequences, or it can simply deliver query responses whenever it receives a message from the NM.

## 6. Performance Evaluation

In this section, we describe our simulation model and evaluate the performance of the proposed management scheme.

### 6.1. Simulation Model

#### 6.1.1. Network Model

Many studies on underwater sensor networks have proposed a cluster-based, vertical tree topology as an energy efficient network figure [29,30]. This is because the vertical tree topology reduces the number of hops required by the data for transmissions to the BS in underwater environments [31]. Thus, we also base our simulation on a cluster-based tree-topology sensor network. We assume that each node has the same number of child nodes, possibly except for the last parent node. Queries uniformly request data transmissions from all sensor nodes, and query responses are aggregated as query result messages are propagated up the tree. Every parent-child pair is assumed to be 1.5 kilometers apart (a second’s distance for an acoustic wave). All queries and response messages are assumed to have average data sizes, and merged query result messages are to have larger sizes than normal query result messages (considering the additional data augmented to the payload). Additional data is added to the response payload as more queries are merged.

#### 6.1.2. Energy Model

Our simulation used LinkQuest Inc.’s UWM1000 model for our underwater acoustic modem energy model. In this model, the payload data rate is 7 kbps, the transmission power 1 Watt, and the reception power 0.75 Watts [32]. Since the energy consumption for sleeping mode and data processing is much less compared to transmission/reception, we do not take these factors into account. We assumed that a node can consume up to 100,000 Joules of energy before it dies out.

#### 6.1.3. Performance Metric

We analyze the average energy consumption (AEC) in terms of joules per node. The average energy consumption is the total network energy consumption divided by the number of sensor nodes in a network. We also observe the energy efficiency rate (EER) defined by the rate of the average energy consumption without merging to that with merging. For example, if the overall energy consumption was 6 kJ without the merging process and 2 kJ with the merging process, the EER is 3.

We also examine network lifetime and network lifetime ratio (NLR), defined by the rate of the network lifetime without the merging and with it. The network lifetime is the time duration until any one node in a network (which will be the sink node in our case since all data is transmitted through the sink node) dies out due to energy depletion.

#### 6.1.4. The Simulation

The parameter for our simulation will be three variables: period range (T), number of queries (Q), and the number of nodes (N). Given a tuple of (T, N, Q), Q queries whose periods are uniformly distributed from 1 up to T are generated and executed for 10,000 time units. We will analyze the sensitivity of the AEC, EER, network lifetime and NLR to (1) the number of queries and (2) the period range.

### 6.2. Simulation Results

In this section, we give an account of our performance results and discuss them.

#### 6.2.1. Sensitivity to the Number of Queries

**Figure 12.**
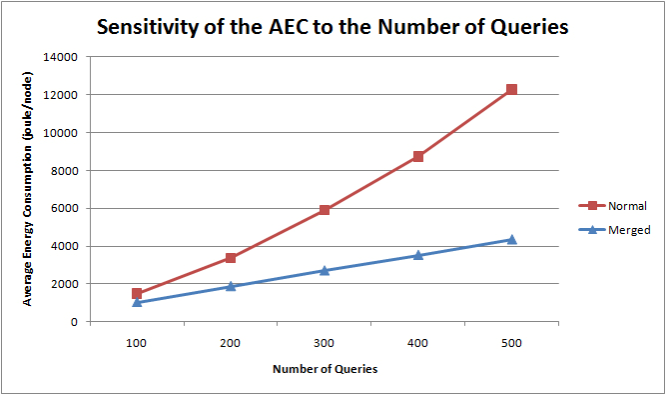
Sensitivity of the AEC to the number of queries.

In the simulation, an increasing number of queries with period range 100 uniformly requested data transmissions from 50 nodes. Figure 12 is the graph of average energy consumption values for each node in a sensor network to the number of total queries.

The graph indicates that with normal data transmission methodologies, the AEC increases slightly more rapidly as more queries are imposed on the whole network. This phenomenon can be attributed to the additional headers attached to the payload for each message transmission and the increased data aggregation at each parent node. In the proposed query management scheme, these redundant data transmissions are minimized and the AEC is kept low. The gradual increase in the AEC is due to the additional fields augmented to the payload at the Network and Data Link layers in response to more queries.

**Figure 13.**
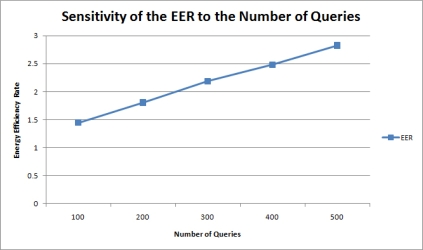
Sensitivity of the EER to the number of queries.

Figure 13 shows the relationship between the number queries and the energy efficiency rate (EER). Performance is improved as more queries were charged on the network. This effect is expectable since the proposed mechanism takes a greater advantage of query result merging when more users query the sensor network.

**Figure 14.**
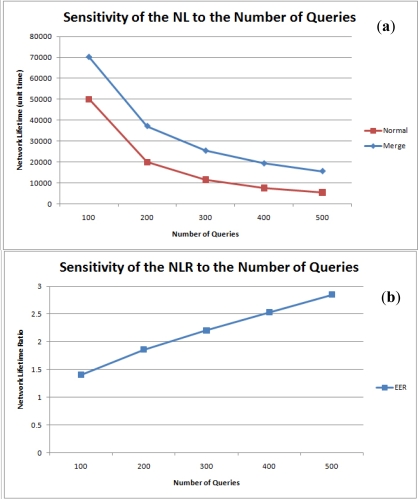
Sensitivity of the **(a)** Network Lifetime and **(b)** NLR to the number of queries.

Figure 14(a,b) are the graphs for network lifetime and the NLR against the number of queries. As expected, network lifetime is inversely proportional to the number of queries and the NLR is proportional to the number of total queries.

#### 6.2.2. Sensitivity to the Period Range

**Figure 15.**
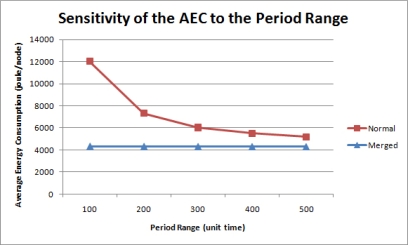
Sensitivity of the AEC to the period range.

**Figure 16.**
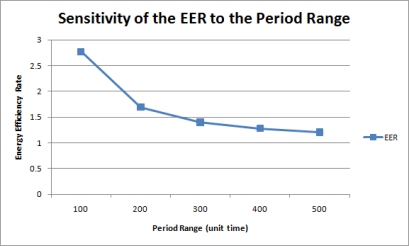
Sensitivity of the EER to the period range.

In this simulation, 500 queries were uniformly imposed on a network of 50 nodes with different period ranges. Figure 15 is the graph of the AEC for each period range. Without merging, the AEC is inversely proportional to the period range because greater query response periods indicate less query execution frequencies. Figure 16 shows the energy efficiency rate for each period range. As expected, the query result merging mechanism becomes less efficient as queries are executed less frequently without much overlapping. Therefore, although the proposed mechanism still proves efficient than the normal query processing methods, its efficiency diminishes as query periods are distributed on a larger period scale.

**Figure 17.**
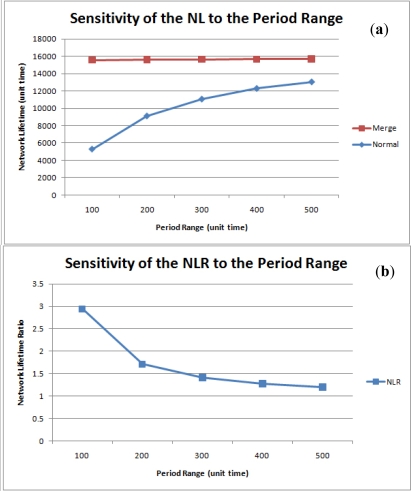
Sensitivity of the **(a)** Network Lifetime and **(b)** NLR to the period range.

Figure 17(a) shows the sensitivity of the network lifetime to the period range. For a normal network, an increase in the period range decreases the number of query executions during the same time interval, so network lifetime in enhanced as the period range is increased. When query results are merged, however, the network lifetime shows only slight variations since each node always produces one tuple at a time. Hence, the NLR is inversely proportional to the number of queries. Nonetheless, query result merging still proves to be efficient in terms of network lifetime, as shown in Figure 17(b).

## 7. Conclusions

In this paper, we have analyzed theoretically query result merging and proposed a new relational database model view and an efficient query management scheme suitable for underwater sensor networks. We also proposed a message payload format capable of maintaining robustness in the face of unexpected communication failures. Through network simulations, we proved how our query management scheme can efficiently reduce redundant message transmissions and the entailed energy waste, and enhance network lifetime. The management mechanism is expected to display higher efficiency when more queries are generated within a relatively smaller period range.

Furthermore, our contributions are not restricted to underwater sensor networks. The proposed methodology may also be applied to terrestrial RF-based sensor networks to significantly reduce network traffic and node energy waste. With the upsurge in the demand for sensor networks as ubiquitous data computing systems, our query result merging framework has great potentials for providing a systematized sensor network management methodology to multiple users.

It is also worth noting that the proposed scheme doesn’t require processing capabilities greater than those of a conventional ubiquitous sensor node. The proposed query models are simple and don’t store data history, thus consuming only a limited space of memory. The query condition merging process in the QRP is also a succession of reasonably arduous logical processes manageable with microprocessors of regular capacity, so the proposed query management scheme asks for no more than the processing capability of a regular sensor node.

There are several ways to improve our research results. One significant direction would be moving the focus from underwater sensor networks to RF-based terrestrial sensor networks and assume reliable time synchronization. Researchers have poured great efforts into theoretical studies for time synchronization, but the current technology is too vulnerable to underwater environments for practical time synchronization to happen. But because time synchronization is easy for terrestrial RF-based sensor networks, adjusting our mechanism to time-synchronized sensor networks for terrestrial applications would bring a great enhancement in sensor network query management in general. Also, developing a method for effectively distributing queries or query sets to the target nodes can significantly enhance the performance of our framework. Lastly, increasing our query functionality by adding other relational operations (the “join” operation, for example) would also be meaningful.
